# Vagus nerve stimulation: a potential tool to further improve perioperative anesthesia management quality and postoperative recovery?

**DOI:** 10.3389/fmed.2026.1721751

**Published:** 2026-03-06

**Authors:** Xiaoyan Dong, Xuejun Sun, Defeng Sun

**Affiliations:** Department of Anesthesiology, The First Affiliated Hospital of Dalian Medical University, Dalian, China

**Keywords:** anesthesiology, autonomic nervous system regulation, pain science, perioperative analgesia, vagus nerve stimulation

## Abstract

Vagus nerve stimulation (VNS) is a promising neuromodulation technique that exerts organ-protective effects through the activation of cholinergic anti-inflammatory pathways and the modulation of the autonomic nervous system. In recent years, its potential applications in perioperative medicine have garnered increasing research attention. A limited body of clinical evidence indicates that VNS can alleviate the inflammatory response induced by surgical stress by inhibiting the α7 nicotinic acetylcholine receptor (α7nAChR)-mediated NF-κB signaling pathway. It may reduce the demand for opioids in perioperative pain management, potentially accelerate patients’ postoperative recovery, and show promise for improving outcomes in cardiac, abdominal, and neurosurgical procedures. Herein, this article explores the potential further application directions of VNS in the perioperative period and summarizes the possible mechanisms based on previous studies, including preoperative anxiety management, intraoperative adjuvant anesthesia, postoperative pain management, and complication prevention. In summary, this review explores the potential of VNS to contribute to personalized perioperative management, while acknowledging that its widespread clinical integration necessitates further rigorous investigation.

## Graphical Abstract

**Figure:**
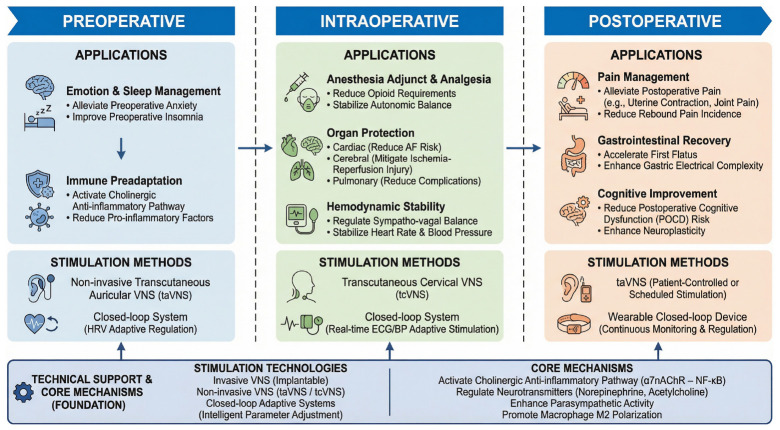
Applications and stimulation methods of vagus nerve stimulation (VNS) in perioperative care: from preoperative preparation to postoperative recovery.

## 1 Introduction

The preoperative, intraoperative, and postoperative phases of the perioperative period constitute a continuous clinical process. Although the Enhanced Recovery After Surgery (ERAS) strategy has been proposed for many years and widely adopted, several areas remain suboptimal. Among them, anxiety regulation, pain management, alleviation of inflammatory stress, and organ function protection remain key challenges in perioperative patient management.

Vagus nerve stimulation (VNS) is a neuromodulation technique that was initially approved for treating epilepsy and depression (1). Currently, the potential of percutaneous vagus nerve stimulation applied to the ear is being extensively investigated for its anti-inflammatory, analgesic, and organ-protective effects. Recent studies indicate that its therapeutic efficacy is comparable to that of invasive vagus nerve stimulation, while the procedure itself is more convenient, facilitating its application during the perioperative period. This article provides a comprehensive review of the existing evidence. It emphasizes recent advancements while also integrating earlier studies that are crucial for understanding the mechanisms underlying VNS. With advancements in VNS detection technology and closed-loop stimulation systems, the objectivity and standardization of VNS applications are anticipated to improve, thereby promoting further research related to perioperative care. This article systematically examines the mechanisms of VNS and its potential applications in perioperative management, focusing on preoperative emotional and sleep regulation, immune preconditioning, intraoperative pain control, organ protection, hemodynamic stability, and postoperative rehabilitation.

## 2 Overview of VNS and closed-loop stimulation systems

### 2.1 Vagus nerve stimulation

#### 2.1.1 Principles and mechanisms

The vagus nerve is among the longest cranial nerves in the human body. It comprises numerous afferent fibers that convey information from various body regions to the brain, while simultaneously allowing the brain to send regulatory signals to multiple organs via the vagus nerve. Vagus nerve stimulation activates these afferent fibers by delivering electrical signals to the nerve, which then transmits the signals to the nucleus tractus solitarius in the brainstem, subsequently influencing brain regions such as the locus coeruleus to regulate neurotransmitter release and achieve therapeutic effects (2).

#### 2.1.2 Stimulation methods and technological advances

Invasive Vagus Nerve Stimulation (iVNS) involves surgically implanting electrodes into the cervical vagus nerve and connecting them to a pulse generator implanted in the chest, suitable for conditions like drug-resistant epilepsy and depression (3). Non-invasive Vagus Nerve Stimulation includes transcutaneous cervical vagus nerve stimulation (tcVNS) and transcutaneous auricular vagus nerve stimulation (taVNS). taVNS stimulates branches of the auricular vagus nerve to regulate central autonomic nervous activity (4). tcVNS, through surface electrodes on the neck skin, can inhibit blood-brain barrier destruction after ischemic stroke, reduce cerebral edema and neurological deficits, and has anti-inflammatory and neurotransmitter-regulating effects (5).

In addition, Schuerman et al. reviewed invasive vagus nerve stimulation and two non-invasive vagus nerve stimulation treatment technologies in 2021. This article will clearly compare the similarities and differences between invasive iVNS, transcutaneous auricular taVNS, and transcutaneous cervical tcVNS in terms of targets, signal transduction pathways, neuromodulator release, application scenarios, etc., and create the following Table 1 (6).

**TABLE 1:** Comparison of invasive and non-invasive vagus nerve stimulation modalities: iVNS, taVNS, and tcVNS.

Comparison aspect	iVNS (invasive)	taVNS (transcutaneous auricular)	tcVNS (transcutaneous cervical)
Stimulation target	Cervical vagus nerve trunk (cervical branch)	Auricular branch of the vagus nerve	Cervical vagus nerve trunk (via surface skin)
Stimulation method	Surgical implantation of a cuff electrode around the cervical vagus nerve	Surface electrodes on the outer ear (e.g., concha, tragus)	Surface electrodes on the neck (near the vagus nerve pathway)
Neural pathway	Direct stimulation of mixed nerve (80% afferent + 20% efferent fibers)	Stimulates purely afferent fibers, with a more complex connectivity	Stimulates afferent fibers, but may be influenced by other cervical nerves (e.g., cervical plexus)
Neuromodulatory release	Activates widespread cortical and limbic systems, promoting NE, ACh, etc., release	May activate similar but weaker or distinct neuromodulatory systems	Similar to taVNS, but due to proximity to iVNS target, may more closely resemble iVNS effects
Application scenarios	Epilepsy, depression, chronic inflammation; neurorehabilitation and cognitive enhancement in trials	Widely studied for mood regulation, pain management, cognitive enhancement, rehabilitation training	Less studied; often explored as a non-invasive alternative to iVNS in clinical trials
Invasiveness/safety	Invasive surgery, risk of infection, nerve injury	Non-invasive, high safety, suitable for long-term or self-administration	Non-invasive, high safety, but may cause skin irritation or discomfort

### 2.2 Closed-loop stimulation system

#### 2.2.1 Concepts and advantages

A Closed-Loop Stimulation System is an intelligent system that can real-time monitor physiological signals and automatically adjust stimulation parameters based on these signals. Through a closed-loop control mechanism, this system integrates stimulation equipment with the body’s physiological regulation system to achieve precise physiological regulation. In the field of neuromodulation, a closed-loop stimulation system usually includes the following key components: ① Sensors: Used to real-time monitor physiological signals, such as electroencephalography (EEG), electrocardiography (ECG), neuronal activity, etc.; ② Control algorithms: Automatically adjust stimulation parameters according to the monitored physiological signals to achieve preset treatment goals; ③ Actuators: Apply electrical stimulation to target nerves or tissues according to the instructions of the control algorithms; ④ Feedback mechanisms: Real-time adjust stimulation parameters by monitoring changes in physiological signals to ensure the stability and effectiveness of treatment effects (7).

#### 2.2.2 Application in VNS

To achieve the optimal efficacy of VNS in the dynamically changing perioperative environment and avoid the uncertainty of fixed-parameter stimulation effects, the closed-loop stimulation system is considered a promising direction for the evolution of VNS technology. In Vagus Nerve Stimulation (VNS), the closed-loop stimulation system can significantly improve treatment effects, reduce side effects, and realize personalized treatment plans by continuously monitoring physiological indicators (such as heart rate, heart rate variability, blood pressure, etc.) and dynamically adjusting stimulation parameters. For example, a new type of taVNS device based on closed-loop biofeedback starts stimulation at specific time points of the cardiac cycle by monitoring photoplethysmography (PPG) signals, thereby achieving more precise physiological regulation (8).

An *in vitro* experiment constructed and verified a battery-free, wireless, closed-loop vagus nerve stimulation (FAW-VNS) system on a pig model: the experiment first confirmed through the open-loop mode that high-frequency and high-duty-cycle stimulation can induce significant bradycardia. Then, in the closed-loop mode, the control unit automatically adjusted down the stimulation parameters according to heart rate feedback, making the heart rate fluctuation converge and stabilize within 2%–4% of the baseline (9).

Although *in vitro* studies cannot be directly extrapolated to the human perioperative period, the results initially suggest that the closed-loop regulation strategy may provide technical feasibility and physiological basis for the safe and individualized implementation of VNS during and after surgery.

## 3 Possible mechanisms of VNS in perioperative medical applications

The therapeutic effects of non-invasive vagus nerve stimulation can be summarized as an overall framework composed of three mechanisms of action. Firstly, the afferent nerve regulation mechanism transmits peripheral stimulation signals to the nucleus tractus solitarius (NTS), which is uploaded through the locus coeruleus-norepinephrine system, thereby regulating the activity of advanced brain networks involved in emotional and cognitive functions. Secondly, by activating the reflexive anti-inflammatory mechanism, the stimulation can trigger the classic “cholinergic anti-inflammatory pathway” reflex arc, thereby inhibiting excessive inflammatory responses at the systemic level. Finally, the efferent nerve regulation mechanism directly regulates peripheral organs through the efferent fibers of the vagus nerve, affecting physiological functions such as heart rate and gastrointestinal peristalsis. These three mechanisms are interrelated and synergistic, jointly forming a multi-level physiological basis for non-invasive vagus nerve stimulation to intervene in neural, immune, and autonomic nervous functions.

### 3.1 Regulating the transmission of neurotransmitters in higher centers

Based on neuroanatomical and preclinical evidence, Goggins et al. proposed that the activation of the auricular branch of the vagus nerve can transmit stimulation information to the nucleus tractus solitarius, and then project to brain regions such as the hypothalamus and hippocampus, promoting adaptive neurochemical changes in the brain, thereby improving patients’ behavior and emotions (10). As stated in the literature review, VNS is postulated to modulate specific neural pathways by regulating the release of neurotransmitters (norepinephrine, acetylcholine, serotonin, γ-aminobutyric acid) in the nuclei, which may contribute to neuroplastic changes in the brain (11). This proposed mechanism finds support in a recent clinical neuroimaging study, which proved that non-invasive vagus nerve stimulation can enhance norepinephrinergic neurotransmission in the human brain, regulate the neural activity of the anterior cingulate cortex, and thereby improve neurocognitive function (12).

In summary, vagus nerve stimulation can effectively promote brain neuroplasticity by activating the nucleus tractus solitarius and regulating the release of key neurotransmitters such as norepinephrine, thereby improving cognitive function and emotional behavior.

### 3.2 Activation of the cholinergic anti-inflammatory pathway (CAP)

#### 3.2.1 α7nAChR - NF-κB signaling axis

The concept of the cholinergic anti-inflammatory pathway was pioneered by Tracey et al. Its core mechanism is that the efferent vagus nerve releases acetylcholine, which acts on α7 nicotinic acetylcholine receptors (α7nAChR) on the surface of immune cells such as macrophages, significantly inhibiting the release of pro-inflammatory cytokines such as TNF and HMGB1, thereby regulating the systemic inflammatory response. This pathway constitutes the efferent link of the “inflammatory reflex” and realizes the rapid regulation of immunity by nerves. Further studies suggest that electrical stimulation of the vagus nerve or central drug intervention can activate this pathway, providing a new theoretical basis and potential intervention strategy for the neuroimmunotherapy of inflammatory diseases (13, 14).

Zhang et al.’s study found that low-level vagus nerve stimulation (LL-VNS) can significantly reduce the levels of TNF-α and IL-6 in serum and atrial tissue, and the level of acetylcholine (ACh) is significantly increased, indicating that LL-VNS inhibits the expression of pro-inflammatory factors by activating α7nAChR, improves the inflammatory microenvironment of the atria, and thereby reduces the inducibility of atrial fibrillation (15). In addition, animal and clinical evidence in the past 2 years have jointly shown that vagus nerve stimulation (VNS) can produce significant neuroprotective effects both in the acute and chronic phases of ischemic stroke: in a mouse model, 60-min low-frequency VNS can reduce infarct volume and improve neurological function regardless of whether it is applied before, during, or after ischemia. The mechanism is to inhibit the assembly of NLRP3 inflammasomes in microglia by activating the α7nAChR pathway, block the release of pro-inflammatory factors such as IL-1β, reduce oxidative stress and apoptosis, thereby rescuing penumbral neurons; a randomized triple-blind controlled trial completed in 19 centers further showed that pairing 0.8 mA, 30 Hz pulsed VNS with upper limb rehabilitation in the chronic phase resulted in a net increase of 2.6 points in the Fugl-Meyer Upper Extremity Score in the intervention group compared with the sham stimulation group, with a low rate of surgery-related adverse events (16, 17).

The above studies consistently reveal that the VNS-α7nAChR-NLRP3 anti-inflammatory and antioxidant pathway is a key molecular target for improving stroke prognosis, providing a translatable new strategy for neuromodulatory intervention in ischemic stroke.

#### 3.2.2 Macrophage phenotype polarization

A recent study that activated the cardiac vagus nerve through optogenetic methods found that it can positively regulate cardiomyocyte proliferation and myocardial regeneration, and activate the IL-10/STAT3 signaling pathway, promoting the polarization of cardiac macrophages to the M2 type, thereby improving cardiac function after myocardial infarction (18). Studies have found that non-invasive vagus nerve stimulation exerts anti-inflammatory and neuroprotective effects by inhibiting the expression of IL-17A and promoting the polarization of microglia/macrophages to the M2 type, providing a new strategy for the treatment of cerebral ischemia-reperfusion injury (19). Therefore, the transformation of macrophage phenotype polarization may play an important role in vagus nerve stimulation.

### 3.3 Regulating the activity of efferent nerves

The efferent fibers of the vagus nerve originate from the dorsal motor nucleus of the vagus nerve in the medulla oblongata and innervate multiple visceral organs. Its main physiological functions include regulating heart rate, gastrointestinal motility, and secretion. For example, acetylcholine released by the vagus nerve terminals causes hyperpolarization of conduction fibers by increasing potassium ion influx, reducing the excitability of excitable tissues, thereby significantly reducing the rhythmic discharge frequency of the sinoatrial node, and then regulating heart rate. In addition, the efferent fibers of the vagus nerve can also regulate gastrointestinal peristalsis through interaction with the enteric nervous system (5). A clinical trial involving 36 patients with functional dyspepsia proved that the dyspepsia symptoms, anxiety and depression scores of patients treated with transcutaneous auricular vagus nerve stimulation were significantly reduced. taVNS activates central autonomic networks such as the dorsal vagal nucleus of the medulla oblongata through the afferent fibers of the auricular branch of the vagus nerve, reflexively enhancing the efferent activity of the vagus nerve, thereby inhibiting the sympathetic tension of the gastric wall, promoting the relaxation of gastric smooth muscle and the synchronization of pacemaker cell rhythm, and ultimately correcting both gastric sensorimotor dysfunction and accompanying emotional disorders (20). An experiment using an *in vivo* ischemia-reperfusion model in pigs proved that vagus nerve stimulation exerts a protective effect against acute myocardial ischemia-reperfusion injury through bilateral muscarinic efferent fibers rather than afferent fibers (21).

### 3.4 Link between integrated mechanisms and perioperative applications

In summary, Vagus Nerve Stimulation (VNS) presents a multifaceted approach to perioperative management through the three interrelated mechanisms previously described. Enhanced central neurotransmitter regulatory mechanisms may provide a neurochemical foundation for alleviating preoperative anxiety while simultaneously improving postoperative cognitive function. The activation of cholinergic anti-inflammatory pathways, employed by VNS for surgical immune preadaptation, mitigates inflammatory responses to surgical stress and facilitates cardiac, cerebral, and pulmonary organ protection. These effects are directly associated with VNS’s potential to modulate the recovery of gastrointestinal function during the postoperative period and to maintain intraoperative hemodynamic stability. The following section will provide a detailed discussion of these specific application scenarios.

## 4 Preoperative applications

### 4.1 Emotion and sleep management

Surgery is a strong stressor, and patients often experience significant psychological and physiological stress responses before surgery (22). Current preoperative sleep and anxiety management still mainly relies on benzodiazepines, antidepressants, or dexmedetomidine, but there are prominent limitations such as respiratory depression, hypotension, bradycardia, and contraindications in elderly patients, prompting the academic community to seek safer solutions. Preliminary studies suggest that non-invasive vagus nerve stimulation may exert sedative and anxiolytic effects by transiently enhancing the parasympathetic nervous system and inhibiting sympathetic hyperactivity, warranting its investigation as a potential complementary approach to perioperative pharmacotherapy (23). Evidence from mechanical rodent models indicates that vagus nerve stimulation (VNS) potentially improves the generalization of conditioned fear extinction and diminishes anxiety. In these studies, VNS markedly reduced responses to non-exposed fear stimuli in rats by enhancing neuroplasticity and modulating neurotransmitter levels. These findings suggest VNS’s potential for providing rapid relief from anxiety and stress in animal models (24). Preliminary clinical findings bolster this notion, indicating that non-invasive auricular vagus nerve stimulation (taVNS) can significantly diminish the fear response in patients with specific phobias, including arachnophobia. The proposed mechanism entails the activation of the norepinephrinergic system and heightened activity in brain regions associated with fear extinction, which in turn inhibits physiological and behavioral responses linked to fear (25). A randomized controlled trial conducted in China, provides more robust evidence, demonstrating that an 8-weeks transcutaneous auricular vagus nerve stimulation (taVNS) intervention significantly alleviated insomnia severity in patients with chronic insomnia. The findings revealed that the Pittsburgh Sleep Quality Index (PSQI) score in the taVNS group decreased by an average of 8.2 points from baseline, indicating a greater reduction of over 4.2 points compared to the sham stimulation group (26). These results offer strong clinical support for the application of taVNS in insomnia management and underscore its potential as a non-pharmacological intervention for preoperative sleep disturbances.

Although the aforementioned studies indicate that VNS may alleviate preoperative anxiety and enhance sleep, potentially offering alternative pharmacological supplements for perioperative management, it is important to note that the existing evidence remains nascent. Most studies are characterized by small sample sizes, heterogeneous research designs, and a lack of large-scale randomized controlled trials focused on specific perioperative populations. The precise therapeutic effects, optimal stimulation parameters (including frequency, intensity, and duration), applicable populations (such as patients of varying ages, types of surgery, and comorbidities), and long-term safety require further investigation. Additionally, the accessibility of VNS devices in the preoperative setting, patient compliance, and cost-effectiveness are practical challenges that must be addressed prior to their integration into routine preoperative preparation.

### 4.2 Surgical immune preconditioning

Existing studies have proven that there is a bidirectional communication mechanism between the nervous system and the immune system, because cells of both systems express common receptors and ligands, through which the nervous system can exert regulatory effects on the immune system, which is also defined as neuroimmunomodulation (27, 28). Based on mechanistic and animal data, a physiological study using a mouse model to investigate the neuroimmunological mechanism of vagus nerve stimulation in inhibiting acute lung inflammation found that selective stimulation of vagus nerve afferent fibers can activate specific regions in the brainstem to promote the adrenal gland to release epinephrine, which then acts on β2-adrenergic receptors on the surface of immune cells in the lung, thereby inhibiting the inflammatory response induced by TLR3 or TLR7 agonists and reducing the release and aggregation of inflammatory factors in alveolar macrophages and neutrophils. This study revealed a new neuroimmune circuit different from the traditional cholinergic anti-inflammatory pathway, proving that the vagus nerve afferent pathway can regulate lung innate immune responses through the adrenal gland-epinephrine-β2AR axis (29).

Although basic and animal studies have consistently demonstrated that vagus nerve stimulation (VNS) downregulates inflammatory factors via cholinergic anti-inflammatory pathways, thereby providing a theoretical foundation for perioperative immunomodulation, these conclusions primarily stem from animal models or *in vitro* investigations. Currently, there is a notable absence of well-designed, large-sample randomized controlled clinical trials to validate the reproducibility and safety of VNS in mitigating inflammatory storms and reducing infections and related complications in actual surgical environments. The translation from animal models to perioperative patients is fraught with challenges, including species differences and variations in disease models and surgical stress. Consequently, the application of VNS for surgical immune preconditioning remains a promising yet inadequately substantiated research avenue. Further systematic clinical studies are essential to clarify its optimal stimulation mode, timing of intervention (e.g., the duration before surgery to initiate treatment), suitable patient populations, and interactions with other perioperative interventions.

## 5 Intraoperative applications

### 5.1 Reducing anesthetic dosage

Vagus nerve stimulation (VNS) can regulate the autonomic nervous system, thereby affecting the patient’s physiological state and alleviating the patient’s stress response to surgery (30). It is hypothesized that this regulatory effect could potentially reduce the demand for anesthetics, because anesthetics are usually used to inhibit stress responses and maintain physiological stability. Based on a single pilot study, a 50-years-old female patient underwent external fixation surgery for open fractures of both the tibia and fibula. Following the surgery, transcutaneous auricular vagus nerve stimulation (taVNS) was employed as an adjunctive analgesic intervention. The results indicated that taVNS significantly decreased the patient’s postoperative pain score (VAS), increased the pressure pain threshold (PPT), and reduced the perioperative consumption of opioids. Furthermore, heart rate variability (HRV) analysis demonstrated that taVNS enhanced parasympathetic nerve activity and improved the balance between sympathetic and parasympathetic responses. This finding suggests that vagus nerve stimulation may effectively alleviate perioperative pain intensity and decrease the requirement for anesthetics by modulating autonomic nervous system function.

However, this remains a highly speculative application. The evidence is confined to a single case report, and the critical leap to intraoperative anesthetic-sparing effects in humans lacks direct clinical support. This hypothesis requires validation in dose-finding RCTs designed specifically to measure volatile or intravenous anesthetic requirements (31).

### 5.2 Regulating perioperative hemodynamics

Perioperative hemodynamic instability is the result of the combined action of multiple factors and mechanisms, involving multiple links such as anesthesia, surgery, the patient’s basic state, and postoperative recovery. Its core mechanisms include insufficient effective circulating blood volume, decreased cardiac output, and tissue hypoperfusion caused by vasodilation (32). Supported by current clinical evidence, recent studies have shown that stellate ganglion block (SGB) can improve cardiac electrophysiological stability, reduce inflammation and stress responses by inhibiting sympathetic nerve activity, thereby helping to maintain perioperative hemodynamic stability, and has shown potential protective effects such as reducing postoperative arrhythmias, stabilizing blood pressure, and reducing the demand for vasopressors in cardiac, thoracic, abdominal, and neurosurgical operations (33). However, as an invasive procedure, SGB still has limitations such as high technical threshold and high risk of complications. Therefore, exploring safer and easier alternative regulatory strategies is of great significance. Non-invasive vagus nerve stimulation (nVNS), a technology that targets autonomic nervous function, is being investigated for regulatory effects that may parallel those of SGB. For example, a recent preliminary study confirmed that low-intensity concha vagus nerve stimulation can effectively inhibit sympathetic nerve activity, regulate the sympathetic-vagal balance, thereby stabilizing heart rate and promoting circulatory system balance (34). Evidence from an animal model showed that right cervical vagus nerve stimulation (cVNS) initiated on the 2nd postoperative day and continued for 6 weeks can significantly improve left ventricular systolic function, reduce the inducibility of ventricular tachycardia and ventricular fibrillation, and inhibit the activation of glia in the stellate ganglion and dorsal root ganglion. This experiment suggests that perioperative non-invasive, titratable cVNS to enhance parasympathetic tone and stabilize myocardial electro-structural heterogeneity is a candidate strategy under investigation for hemodynamic regulation, but its clinical dosage and timing still need further verification (35). Further supporting this potential, a randomized controlled trial involving patients with postural tachycardia syndrome (POTS) proved that transcutaneous auricular vagus nerve stimulation (tVNS) significantly enhanced hemodynamic stability by reducing the activity of anti-adrenergic autoantibodies β1AR and α1AR, inflammatory factor TNF-α, and improving heart rate variability (36). A small pilot study involving five healthy volunteers demonstrated that non-invasive tragus vagus nerve stimulation (aVNS) at parameters of 25 Hz and 200 μs resulted in an increase in the high-frequency component of heart rate variability and a 64% reduction in the LF/HF ratio. These findings suggest a rapid enhancement of parasympathetic activity. The preliminary results indicate that trans-tragus aVNS can quickly reshape autonomic balance, offering a promising approach for the rapid and non-invasive optimization of hemodynamics during the perioperative period. However, further investigation with larger sample sizes is necessary to verify its efficacy and establish safe dosage guidelines (37).

Although the aforementioned preclinical and preliminary clinical studies indicate the potential of vagus nerve stimulation (VNS) to modulate autonomic homeostasis and stabilize heart rate and hemodynamics, its efficacy and safety within the complex and variable perioperative environment remain unestablished. Existing clinical studies have primarily concentrated on specific pathological states (e.g., POTS) or healthy volunteers, and the sample sizes are generally small. In perioperative patients with multiple comorbidities who are undergoing surgical trauma and experiencing the effects of anesthetic agents, the cardiovascular effects of VNS may be unpredictable and could even pose risks of inducing bradycardia, hypotension, or arrhythmia. Currently, there is an absence of recognized safe and effective stimulation parameter regimens tailored for different types of surgery and patient populations. To potentially improve the routine application of VNS in perioperative hemodynamic management, it is essential to first delineate its efficacy boundaries and risk profile through a large-scale, multicenter randomized controlled trial (RCT).

### 5.3 Organ protection during surgery

Supported by clinical research, a study on autonomic nerve modulation therapies (ANMTs) for preventing postoperative atrial fibrillation (POAF) after cardiac surgery found that interventions such as low-level vagus nerve stimulation (LL-VNS) and epicardial injection (e.g., botulinum toxin or calcium chloride) can inhibit excessive sympathetic and parasympathetic activation, prolong atrial effective refractory period, and reduce inflammatory factor IL-6 levels, thereby decreasing the incidence (OR: 0.37; 95% CI: 0.25–0.55) and burden (mean reduction: 3.51 h) of POAF and shortening hospital stay (mean reduction: 0.82 days) (38). In contrast, preclinical evidence from rodent models indicates that transcutaneous auricular vagus nerve stimulation (ta-VNS) exerts protective effects against cerebral ischemia–reperfusion injury by regulating acetylcholine levels, inhibiting inflammatory factor secretion, and suppressing phosphorylation of connexin 43 (Cx43). This mechanism involves activation of the cholinergic anti-inflammatory pathway and modulation of intercellular communication, providing a theoretical basis for the potential use of VNS in perioperative neuroprotection (39). Similarly, experimental animal studies have demonstrated that VNS can reduce postoperative pulmonary complications following esophagectomy. In a rat model simulating intraoperative vagal transection, VNS significantly attenuated LPS-induced pulmonary neutrophil infiltration (VNS-50 group: 19 ± 15 × 104, *P* = 0.003; VNS-10 group: 15 ± 6 × 104, *P* = 0.009) and alleviated lung tissue injury (LIS score in VNS-50 group: 0.316 ± 0.093, *P* = 0.043; unilateral VNS-50 group: 0.296 ± 0.065, *P* = 0.005). The protective effect depends on vagal integrity and activation of the cholinergic anti-inflammatory pathway, suggesting that VNS may mitigate pulmonary complications after esophagectomy, although stimulation parameters and potential adverse effects require further clinical evaluation (40).

The diverse mechanistic potential of vagus nerve stimulation (VNS) in protecting multiple organs, including the heart, brain, and lungs, is promising. However, it is important to acknowledge that high-quality clinical evidence supporting its efficacy in human perioperative organ protection remains exceedingly limited. Most conclusions are drawn from animal studies, which cannot fully replicate the complexities of human surgery or the variability in patient conditions. For instance, research aimed at preventing atrial fibrillation during cardiac surgery necessitates larger-scale clinical validation. Furthermore, intraoperative applications encounter significant technical integration challenges. These include ensuring the safe placement and operation of the stimulation device within a sterile environment, avoiding electromagnetic interference with surgical instruments and monitoring equipment, and customizing stimulation parameters for various organ protection targets. Addressing these issues will depend on the collaborative advancement of future engineering innovations and the design of rigorous clinical studies.

## 6 Postoperative applications

### 6.1 Pain management

Supported by a randomized clinical trial involving 156 parturients undergoing elective cesarean section, the effect of transcutaneous auricular point vagus nerve stimulation (taVNS) on relieving postpartum uterine contraction pain was investigated. The study demonstrated that taVNS can significantly reduce the incidence of moderate to severe uterine contraction pain on the third postoperative day, while also alleviating incision pain, improving postpartum anxiety and depression, and enhancing recovery quality and sleep quality. These findings confirm that taVNS is a safe, effective, and novel strategy to promote postoperative rehabilitation after cesarean section (41). Further supporting its role in pain management, another randomized controlled trial evaluated the effect of transcutaneous auricular vagus nerve stimulation (taVNS) on postoperative rebound pain in patients undergoing anterior cruciate ligament reconstruction (ACLR). The results showed that both the incidence and duration of rebound pain were significantly lower in the taVNS group compared with the sham stimulation group. It is hypothesized that taVNS may reduce postoperative pain by inhibiting neuronal discharge and modulating inflammatory responses (42).

Despite the promising potential of Vagus Nerve Stimulation (VNS) as a non-pharmacological adjuvant analgesic strategy, significant limitations in its current clinical application hinder its widespread adoption. First, the lack of standardization in technical parameters presents a challenge; key factors such as stimulation frequency, intensity, waveform, and treatment duration vary considerably across studies, complicating the reproducibility of efficacy and obstructing the determination of optimal treatment regimens. Second, the quality of clinical evidence requires enhancement; most existing positive findings originate from studies with small sample sizes focused on specific surgical procedures (e.g., cesarean section), which introduces a general risk of bias. There is a notable absence of high-quality, large-sample, multicenter randomized controlled trials (RCTs) that validate VNS effectiveness across diverse surgical types and elucidate potential synergistic effects with established multimodal analgesic regimens. Finally, the mechanisms that account for variations in individual responses remain unclear, making the identification of patient populations most likely to benefit from VNS a critical area for future research.

### 6.2 Improving postoperative gastrointestinal function

Postoperative Gastrointestinal Dysfunction (POGD) is a prevalent and clinically significant complication following surgical procedures. It adversely impacts patient comfort, extends the duration of postoperative hospital stays, and elevates the risk of perioperative complications and unplanned readmissions, ultimately resulting in increased medical costs (43). A randomized double-blind controlled trial involving 22 patients undergoing gastrointestinal surgery demonstrated that those receiving 15 min of transcutaneous auricular vagus nerve stimulation (taVNS) daily until the occurrence of the first flatus experienced a significantly shorter time to first flatus compared to the sham stimulation group. Additionally, electrogastrography analysis indicated that taVNS significantly enhances the complexity and rhythm variability of normal gastric slow waves without altering their power ratio. This finding confirms that taVNS effectively promotes the recovery of postoperative gastrointestinal function by modulating the dynamic complexity of gastric myoelectric activity (44). Findings across trials have demonstrated inconsistency. One randomized controlled trial confirmed that non-invasive vagus nerve stimulation can enhance intestinal smooth muscle function via the vagus nerve’s anti-inflammatory pathway in patients following colorectal surgery; however, it did not establish a significant reduction in the time required for postoperative intestinal function recovery. The authors concluded that larger-scale definitive clinical trials are necessary to assess its precise clinical efficacy (45). Additionally, a controlled trial involving a specific patient population at three Steno Diabetes Centers in Denmark revealed that among 145 diabetic patients with autonomic neuropathy undergoing lateral cervical transcutaneous vagus nerve stimulation, there was no statistically significant difference in gastrointestinal function improvement between the tVNS group and the sham stimulation group, nor was there an enhancement in cardiovascular autonomic function. This indicates that this particular intervention did not alleviate the symptoms associated with diabetic gastrointestinal lesions through the vagal-cholinergic mechanism (46).

In summary, VNS has been explored as a novel therapeutic approach for improving gastrointestinal dysfunction, but the available clinical evidence is contradictory and inconsistent. This heterogeneity in efficacy may stem from a number of factors: differences in stimulation targets and parameters (e.g., ear vs. neck, different frequencies/intensities), different pathophysiologic states of the study populations (e.g., postoperative intestinal obstruction vs. diabetic autonomic neuropathy), and variable sensitivities in the assessment of endpoints. The negative results of the Danish study are a particular reminder that the efficacy of VNS is not universal and that its effects are highly dependent on whether the underlying pathologic mechanisms involve vagal-cholinergic pathways that can be modulated by VNS. Therefore, future studies need to work on identifying specific patient subtypes that may benefit from VNS and precisely optimizing the stimulation regimen, rather than viewing it as a generic gastrointestinal function enhancer.

### 6.3 Improving cognitive impairment

Perioperative brain injury mainly focuses on two categories: the first category includes stroke, transient ischemic attack, and coma; the second category mainly includes cognitive function changes and epileptic seizures. Perioperative brain injury is mainly dominated by the second category. Perioperative neurological complications may increase the mortality rate of patients (47). Preclinical evidence from animal models suggests that vagus nerve stimulation (VNS) can alleviate manifestations akin to postoperative cognitive dysfunction (POCD). In a murine study examining the effects of VNS, researchers found that VNS significantly enhances auditory perceptual learning by activating the central cholinergic system. This enhancement occurs through increased cortical plasticity, indicating that VNS may improve cognitive function by modulating the neuromodulator systems within the central nervous system (48). Additional mechanistic insights from rodent studies reveal that a single 30-min VNS session in healthy adult male rats can markedly improve cognitive performance. This improvement is evidenced by longer interaction times and higher exploration frequencies with novel objects in the Novel Object Recognition (NOR) task, as well as a delayed latency to enter the dark compartment in the Passive Avoidance Task (PAT). Mechanistically, researchers propose that VNS enhances cognitive function by promoting synaptic plasticity in the hippocampal CA1 region, as evidenced by increased Long-Term Potentiation (LTP), elevated amplitude and frequency of spontaneous excitatory postsynaptic currents, and upregulated expression of Brain-Derived Neurotrophic Factor (BDNF) in the CA1 and CA2 regions (49). A multimodal exploratory study that integrated behavioral tests, functional Near-Infrared Spectroscopy (fNIRS), and virtual brain simulation systematically examined the regulatory effects and neural mechanisms of transcutaneous auricular vagus nerve stimulation (taVNS) on the working memory of elderly individuals. The findings indicated that the cognitive enhancement induced by taVNS is not universally applicable; instead, its effects and direction are contingent upon the individual’s pre-intervention brain network structure and functional state. This provides critical theoretical and computational insights for the development of individualized and precise neuromodulation strategies (50). In a randomized controlled clinical trial assessing the impact of taVNS on cognitive function in patients with Mild Cognitive Impairment (MCI), it was demonstrated that taVNS significantly improved patients’ total scores on the Montreal Cognitive Assessment-Basic (MoCA-B). This supports the hypothesis that vagus nerve stimulation enhances cognitive function by regulating neural networks and neurotransmitter levels, thereby offering a compelling rationale for its potential role in alleviating perioperative cognitive dysfunction (51). Further underscoring its clinical significance, a recent interventional study revealed that administering taVNS 1 h prior to anesthesia induction and continuing until the conclusion of surgery can reduce the incidence of delayed neurocognitive recovery in elderly patients following total joint replacement (52). In summary, vagus nerve stimulation (VNS) and its non-invasive forms, such as taVNS, represent promising avenues for cognitive enhancement and recovery.

Vagus nerve stimulation (VNS) and its non-invasive variants have demonstrated mechanistic plausibility and initial clinical evidence suggesting improvements in cognitive function and a delay in postoperative cognitive decline. Nonetheless, their application is complex and not straightforward. First, significant individual variability in efficacy exists, with studies indicating that the effects of VNS depend on the pre-intervention state and structure of the individual’s brain network. This variability suggests that a “one-size-fits-all” approach may prove ineffective. Second, the optimal stimulation parameters remain undefined. Specifically, the ideal timing for stimulation–whether preoperative, intraoperative, or postoperative–along with the appropriate frequency, intensity, and duration of treatment, is still inconclusive. Additionally, the long-term safety and underlying neural circuit mechanisms are not well understood; the potential long-term effects of repeated or prolonged stimulation on complex neural networks have yet to be thoroughly examined. Furthermore, its current application is confined to exploratory adjunctive interventions and cannot substitute for fundamental perioperative brain protection measures. The translation of these findings into standardized protocols necessitates navigating the extensive journey from individualized mechanistic insights to large-scale clinical validation.

To more systematically and intuitively assess the application prospects and evidence base of vagus nerve stimulation across various perioperative phases, this review compiles and summarizes the available literature based on primary study types, sample characteristics, and key findings into the following Table 2.

**TABLE 2:** Summary of current evidence for peri-operative applications of vagus-nerve stimulation.

Timing	Clinical field	Modality	Target population	Key findings/effects	Study design	References
Pre-operative	Mood and sleep	VNS	Rat model	Rapidly alleviated anxiety and stress in rats	*In vivo*	(24)
		taVNS	Arachnophobic patients	Reduced anxiety and stress levels in phobia patients	RCT	(25)
		taVNS	Chronic insomnia patients	Alleviated insomnia symptoms	RCT	(26)
	Immune priming	VNS	Mouse pneumonia model	Vagal afferents down-regulated pulmonary innate immunity via adrenal-epinephrine-β2AR axis	*In vivo*	(29)
Intra-operative	Anesthetic reduction	taVNS	Fracture surgery patients	Significantly reduced postoperative pain scores (VAS), increased pressure pain threshold (PPT), and decreased perioperative opioid use	Case series	(31)
	Hemodynamic control	Stellate ganglion block (SGB)	Cardiac, thoracic, abdominal and neurosurgery patients	Improved cardiac electrophysiological stability, maintained perioperative hemodynamic stability, reduced postoperative arrhythmias	Systematic review	(33)
		LL-aVNS	Paroxysmal atrial fibrillation patients	Stabilized heart rate and promoted circulatory balance	RCT	(34)
		cVNS	Mini-pig myocardial infarction model	Improved left ventricular systolic function, reduced ventricular tachycardia and fibrillation inducibility	*In vivo*	(35)
		tVNS	Postural orthostatic tachycardia syndrome (POTS) patients	Improved heart rate variability and enhanced hemodynamic stability	RCT	(36)
		aVNS	Healthy volunteers	Rebalanced autonomic tone within minutes	RCT	(37)
	Organ protection	Autonomic neuromodulation therapy (ANMT), i.e., LL-VNS combined with epicardial injection	Cardiac surgery patients	Reduced incidence of postoperative atrial fibrillation (POAF)	RCT	(38)
		taVNS	Cerebral ischemia-reperfusion injury rats	Significantly improved neurological recovery in rats	*In vivo*	(39)
		VNS	Rat esophagectomy model	Reduced postoperative pulmonary complications after esophagectomy	*In vivo*	(40)
Post-operative	Pain management	taVNS	Cesarean delivery mothers	Significantly reduced incidence of moderate-to-severe uterine cramping pain on postoperative day 3 and alleviated incisional pain	RCT	(41)
		taVNS	Anterior cruciate ligament reconstruction (ACLR) patients	Attenuated rebound pain by suppressing nerve discharge and modulating inflammatory response	RCT	(42)
	Gastrointestinal function regulation	taVNS	Gastrointestinal surgery patients	Regulated gastric myoelectric activity and reduced time to first flatus	RCT	(44)
		nVNS	Colorectal surgery patients	Improved intestinal smooth muscle function, but did not significantly shorten bowel function recovery time	RCT	(45)
		tVNS	Diabetic patients with autonomic neuropathy	Did not alleviate diabetic gastrointestinal symptoms via vagal-cholinergic mechanisms	RCT	(46)
	Cognitive improvement	VNS	Mouse model	Improved cognitive function by modulating central nervous system neuromodulator systems	*In vivo*	(48)
		VNS	Healthy adult male rats	Enhanced synaptic plasticity in hippocampal CA1 region to achieve cognitive improvement	*In vivo*	(49)
		taVNS	Elderly individuals	Cognitive improvement not universal; effect and direction depend on individual baseline brain network structure and functional state	RCT	(50)
		taVNS	Mild cognitive impairment patients	Improved cognitive function by modulating neural networks and neurotransmitter levels	RCT	(51)
		taVNS	Elderly patients undergoing joint replacement surgery	Reduced incidence of delayed neurocognitive recovery	RCT	(52)

## 7 Challenges, risks, and future directions in clinical translation

As an intervention strategy with multi-target regulatory potential, vagus nerve stimulation has demonstrated promise in preclinical and early clinical studies for managing anxiety, pain, and inflammation, as well as promoting organ protection in perioperative medicine. However, its translation into routine perioperative care remains constrained by several core challenges that must be systematically addressed.

Firstly, the clinical evidence remains limited, and the technical parameters lack standardization. Most current evidence supporting perioperative applications, such as organ protection and anesthetic-sparing effects, derives from animal studies, with a notable deficiency of high-quality randomized controlled trials (RCTs). Key stimulation parameters, including frequency, intensity, waveform, and duration, exhibit considerable variability across studies, resulting in heterogeneous outcomes and impeding the establishment of optimized treatment protocols. Although safety data for non-invasive methods, such as taVNS, indicate that serious complications are rare and adverse events, including local skin irritation and transient headache, are generally mild and reversible, these safety profiles require further validation within the complex and dynamic perioperative environment.

Secondly, significant practical barriers hinder clinical implementation, extending beyond the general acknowledgment of “technical challenges.” ① Sterility and device integration: Intraoperative application must align stimulation with the sterile surgical field. Emerging practical solutions include the use of single-use, sterile auricular electrodes applied by the anesthesia team in the pre-operative holding area before skin preparation. Additionally, wireless, compact taVNS devices with disposable interfaces are under development to reduce interference with surgical drapes and monitoring lines. Electromagnetic compatibility with electrocautery and other equipment remains a concern; future designs may necessitate shielding or synchronized operation protocols to prevent interference. ② Workflow integration: The feasibility of implementing taVNS varies throughout the perioperative pathway. In the pre-operative holding area, it could be utilized for anxiety and sleep modulation, potentially decreasing the need for pharmacological premedication. In the post-anesthesia care unit (PACU), taVNS may act as an adjunct for pain management and hemodynamic stabilization. On the surgical ward, daily sessions could facilitate gastrointestinal recovery and cognitive rehabilitation. A phased, protocol-driven approach–tailored to the type of surgery and patient-specific risks–would optimize resource utilization and adherence. ③ Training and Protocol Development: The successful adoption of new practices relies on structured training programs for anesthesiologists, nurses, and perioperative technicians. Essential components include device operation and troubleshooting, patient selection criteria–such as the exclusion of individuals with cervical vascular anomalies or local skin lesions–integration into existing enhanced recovery pathways, and thorough documentation and outcome tracking. The development of protocols should involve multidisciplinary collaboration among anesthesiology, surgery, nursing, and biomedical engineering to ensure safe and effective implementation. ④ Cost-Effectiveness and Device Accessibility: The economic implications of routine vagus nerve stimulation (VNS) use remain uncertain. Initial device costs, maintenance, and staff training expenses must be evaluated against potential reductions in opioid consumption, shorter hospital stays, and fewer complications. Furthermore, for patients with implanted VNS devices, such as those for epilepsy, perioperative management currently lacks unified guidelines and necessitates specific planning to prevent electromagnetic interference from surgical equipment.

The physiological effects of vagus nerve stimulation (VNS) present uncertain risks and management complexities. In patients experiencing hemodynamic instability or severe respiratory disease, VNS may provoke unpredictable cardiovascular fluctuations or airway responses, thereby increasing perioperative risk. The safe application of VNS relies heavily on individualized treatment plans, ongoing patient education, and effective multidisciplinary coordination. This complexity can create a management bottleneck in emergency situations, hindering broader adoption. Future directions should focus on the mechanistic clarification of neuro-immune pathways, the conduct of high-quality prospective clinical trials to strengthen the evidence base, technological innovations such as anti-interference designs and intelligent closed-loop systems, and the development of standardized multidisciplinary management frameworks. Through coordinated efforts in basic research, clinical validation, and engineering optimization, VNS may be integrated more safely and effectively into perioperative care systems, ultimately enhancing patient outcomes and reshaping recovery trajectories (53).
